# Genome Sequence of *Sporolactobacillus terrae* DSM 11697, the Type Strain of the Species

**DOI:** 10.1128/genomeA.00465-14

**Published:** 2014-05-29

**Authors:** Kaiming Huang, Jun Ni, Ke Xu, Hongzhi Tang, Fei Tao, Ping Xu

**Affiliations:** State Key Laboratory of Microbial Metabolism & School of Life Sciences and Biotechnology, Shanghai Jiao Tong University, Shanghai, China

## Abstract

*Sporolactobacillus terrae* DSM 11697 is the type strain of *S. terrae*. Here, we present a 3.2-Mb assembly of its genome sequence. As *S. terrae* is one of the important lactic acid bacteria, the genome sequence may provide insights into the molecular mechanism for its further microbial investigation.

## GENOME ANNOUNCEMENT

Members of the genus *Sporolactobacillus* have been defined as catalase-negative, spore-forming, homofermentative, lactic acid-producing organisms that belong to the family *Lactobacillaceae* (1). Six species in the genus *Sporolactobacillus* have been reported, including *S. inulinus*, *S. kofuensis*, *S. lactosus*, *S. laevolacticus*, *S. nakayamae*, and *S. terrae* (2). Members of the genus *Sporolactobacillus* are known for their high-optical-purity lactic acid-producing capabilities; thus, they have been suggested to be good producers of lactic acid (3). Lactic acid is widely used in the food, pharmaceutical, textile, and leather industries; moreover, it is a building block for a biodegradable plastic, polylactic acid (4, 5).

As the type strain of *S. terrae*, strain DSM 11697 produces the acid from maltose, inulin, mannose, trehalose, and galactose, but not from raffinose (6). On the contrary, a different *S. terrae* strain, HKM-1, was newly isolated, which can produce the acid from raffinose but not from the other five carbon sources. To better understand the biochemical and physiological differences of these two *S. terrae* strains, we sequenced the genome of strain DSM 11697.

The draft genome sequence of *S. terrae* DSM 11697 was obtained using the Illumina GA system. Sequencing was performed by the Chinese National Human Genome Center at Shanghai, China, with a paired-end library. The reads were assembled using the Velvet software (7). The genome was annotated using the Rapid Annotations using Subsystems Technology (RAST) automated annotation server (8). The G+C content was calculated using the genome sequence. The functional description was determined by using Clusters of Orthologous Genes (9). rRNA and tRNA genes were identified by RNAmmer 1.2 (10) and tRNAscan-SE (11), respectively.

The genome sequence of DSM 11697 has a G+C content of 46.03%. The number of contigs (>100 bp) is 102, and the number of bases is 3,204,401. There are 3,386 putative coding sequences (CDSs) (818 bp average length), with 3,310 CDSs having functional predictions, 63 tRNA genes, and 7 rRNA operons in the genome sequence.

There are 377 subsystems represented in the genome sequence. The genes encoding proteins responsible for the production of lactic acid were successfully annotated. The *gyrB* gene encodes subunit B of gyrase and is distributed universally in all bacteria, with an average substitution rate of 0.7% to 0.8% per million years (12). We also annotated the *gyrB* gene of *S. terrae* DSM 11697 and confirmed it by PCR and sequencing. Although there are no differences between the 16S rRNA genes of *S. terrae* DSM 11697 and *S. terrae* HKM-1, according to sequence alignment, there are two distinct bases in the *gyrB* gene. The obtained genome sequence provides weighty evidence to distinguish *S. terrae* DSM 11697 and *S. terrae* HKM-1, as well as useful information for their further microbial investigation.

### Nucleotide sequence accession numbers.

This whole-genome shotgun project has been deposited at DDBJ/EMBL/GenBank under the accession no. JFZC00000000. The version described in this paper is the first version, with accession no. JFZC01000000.
